# Quantification of Thyroid Viscosity in Healthy Subjects Using Ultrasound Shear Wave Dispersion (Viscosity PLUS)

**DOI:** 10.3390/diagnostics12092194

**Published:** 2022-09-09

**Authors:** Dana Stoian, Luciana Moisa, Laura Taban, Ioan Sporea, Alexandru Popa, Felix Bende, Alina Popescu, Andreea Borlea

**Affiliations:** 1 Division of Endocrinology, Department of Internal Medicine II, “Victor Babes” University of Medicine and Pharmacy, E. Murgu Square, Nr. 2, 300041 Timisoara, Romania; 2 DrD Ultrasound Center, M. Cristea Nr. 9, 300029 Timisoara, Romania; 3 Division of Gastroenterology and Hepatology, Department of Internal Medicine II, Center for Advanced Research in Gastroenterology and Hepatology, “Victor Babes” University of Medicine and Pharmacy, E. Murgu Square, Nr. 2, 300041 Timisoara, Romania

**Keywords:** viscosity, ultrasound, normal thyroid, Vi PLUS, 2D SWE, shear wave dispersion, thyroid elastography

## Abstract

Shear-wave elastography (SWE) is widely used in thyroid evaluation, but multiple factors influence thyroid stiffness. Estimating tissue viscosity may enhance the ultrasound diagnosis of thyroid diseases, along with the ultrasound (US) and the SWE assessment. In order to be able to detect diffuse thyroid disease by viscosity measurements, it is essential to firstly define the normal values of thyroid viscosity in healthy subjects. Currently there are no published data on thyroid viscosity measurements. This first prospective study aimed to determine the normal range of thyroid viscosity values in a cohort of healthy thyroids, as well as to determine the factors that may influence them. One hundred and twenty-one consecutive subjects without thyroid pathology were evaluated in the study by means of conventional ultrasound, two-dimensional SWE (2D SWE PLUS) and viscosity plane-wave ultrasound (ViPLUS) embedded in the Supersonic MACH^®^ 30 ultrasound system. Five valid tissue viscosity measurements were obtained for each thyroid lobe in every patient and the median values were analyzed and correlated with the biological and demographic parameters of each patient. Our results reveal that ViPLUS is a highly feasible and reproducible technique for thyroid evaluation. Thyroid stiffness, age, gender, BMI and depth of measurements did not influence the thyroid viscosity values. The mean thyroid viscosity by ViPLUS for normal thyroid tissue was of 2.42 ± 0.41 Pa·s. Viscosity assessment by Supersonic ViPLUS is an innovative, non-invasive technique that has proven to be useful for thyroid US evaluation and remains to demonstrate its effectiveness in identifying patients with thyroid disease.

## 1. Introduction 

Thyroid ultrasound (US) is the preferred evaluation for thyroid morphology. It is used to examine the size, echogenicity, homogeneity, vascularization of the parenchyma, and also to detect the presence and appearance of thyroid nodules [1,2]. When compared to clinical examination, it leads to much improved estimates of different thyroid pathologies, as reported by epidemiological studies [3]. Given the excellent diagnostic performance of US evaluation in detecting thyroid disease, additional US-based techniques have been intensely studied in recent years [4]. Ultrasound-based elastography techniques have gained widespread acceptance for their evaluation of liver fibrosis [5,6] and for their ability to examine superficial structures such as the thyroid [7,8] or breast [9,10,11]. Because of this, tissue stiffness has emerged as a novel biomarker in clinical practice. Strain elastography (SE) determines the stiffness of the tissue by exerting external tissue pressure. Two-dimensional (2D) shear-wave elastography (SWE) provides an in vivo, non-invasive estimate of soft tissue stiffness based on the propagation velocity of shear-waves through tissues [12]. It has been demonstrated to be a sensitive imaging technique that can identify diffuse thyroid disease such as Hashimoto’s thyroiditis [13] and can distinguish between benign and malignant thyroid nodules [7,14,15,16,17]. However, it is recommended that 2D-SWE evaluation of the thyroid always accompanies and combines the B-mode ultrasound [18].

Up until now, with the use of US SWE methods, researchers have assumed that the evaluated structures are elastic, linearly uniform, and isotropic [19], but biological soft tissues are naturally viscoelastic and not completely elastic [20]. Due to technological and imaging advancements in the field of biomechanics, there is growing evidence of the diagnostic utility of elastic indices other than linear stiffness [21]. The shear wave dispersion mechanism, which is linked to inflammatory changes in tissues, is the basis for this novel imaging parameter: viscosity. Sugimoto et al. claim, in their study, that an increase in tissue necro-inflammatory alterations corresponds to an increase in dispersion, resulting in higher values of tissue viscosity [22]. This new imaging tool may eventually play a significant role in clinical elastographic diagnosis [21]. 

In diffuse thyroid pathology, viscosity may help differentiate between inflammation and fibrosis and speed up the diagnosis process. Moreover, this tool could potentially be helpful in identifying thyroid cancer. Previous studies have demonstrated that cancerous and normal cells interact with the neighboring tissue differently and have particular viscoelastic properties [23].

Research on viscosity as an imaging parameter is still at an early stage. In order to distinguish diffuse thyroid diseases from healthy parenchyma, normal thyroid viscosity values and their variability need to be defined; to our knowledge, there are no data currently published in this regard. The aim of this study was to determine the feasibility of the method (viscosity plane-wave ultrasound), the range of values for thyroid viscosity in a healthy cohort and to assess the influences of age, gender and body mass index (BMI) on this parameter.

## 2. Materials and Methods

### 2.1. Study Population

A prospective monocentric study was conducted for two months (March–April 2022). One hundred and twenty-one consecutive subjects were enrolled, all of whom had normal values of the thyroid stimulating hormone (TSH), negative familial history of thyroid pathology and normal appearance of the thyroid parenchyma at US examination. In all cases, a multiparametric US evaluation was performed within each session which included: B-mode, 2D-SWE and viscosity measurements.

The exclusion criteria were: subjects with known medical history of thyroid disease, with personal history of head or neck radiation therapy for neoplasia, subjects with personal history of any autoimmune disease or known relatives with chronic thyroid disease. All patients resided in Timis County, Romania, which is historically considered an iodine replete area [24].

The study was carried out in accordance with the Declaration of Helsinki, amended in 2000, Edinburgh, and was authorized by the Local Ethics Committee of the County Emergency Hospital “Pius Brinzeu” Timisoara (nr. 235/2021). Prior to study enrollment, all participants provided their written informed consent.

### 2.2. Two-Dimensional SWE PLUS and Viscosity PLUS Measurements

For the ultrasound examination, the patient was placed in a supine position, with hyperextension of the neck, using coupling gel between the transducer and the skin of the neck. A US operator with more than 4 years of experience in SWE of the thyroid (D.S.) performed all examinations. 

The evaluation was performed using the innovative SuperSonic MACH^®^ 30 ultrasound system (Supersonic Imagine, Aix-en-Provence, France) with the UltraFast™ software for image acquisition. The depth was adjusted so as to include the entire thyroid lobe in the center of the image on the display in B-mode and the measurements were performed by visualizing the thyroid lobe in longitudinal plane. 

For tissue viscosity and shear-wave elastography assessment, the 2D ShearWave (2D-SWE PLUS) mode was activated in conjunction with the Viscosity Plane-wave UltraSound (ViPLUS) mode, available on the curvilinear C6-1X transducer, which was used for measurements. ViPLUS is an extra parameter that may be obtained along with the 2D-SWE measurements. The shear waves’ velocity variations between different frequencies are displayed in the form of a color-map (white-yellow-red), but the machine also provides a numerical value, expressed in pascal-seconds (Pa·s). The machine is set to offer values in a range from 1.0 to 5.0 Pa·s. For 2D-SWE PLUS, a colored map of tissue stiffness is displayed in parallel with the Vi PLUS picture, using ultrafast imaging methods. The color scale goes from dark blue–yellow–dark red, representing Young’s modulus values (kPa). A quantitative value of tissue elasticity is also provided, measured in kPa.

The SWE/ViPLUS acquisition box was positioned so as to include the thyroid lobe and the region of interest (ROI or QBox™) was placed in an area where the color-map is completely colored and shows no artifacts. Measurements were made after a 5 s image stabilization and with a stable color-coded map on the device’s screen. The diameter of the Qbox was set between 5 and 10 mm. A valid assessment was defined as measurements obtained with a fully colored QBox and a stability index (SI) above 90%, which is a new quality parameter developed by SuperSonic Imagine. Five valid measurements were obtained from 5 different frames for thyroid stiffness (2D SWE PLUS) and also for thyroid viscosity (ViPLUS) for each thyroid lobe, in every subject. The median values for each lobe were obtained and correlated with the clinical and demographic parameters of each patient. 

**Figure 1:**
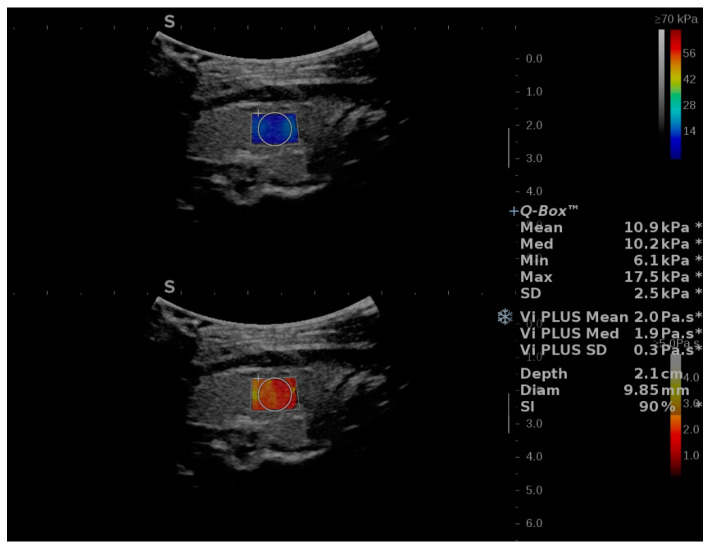
Illustration of two-dimensional Shear-Wave Elastography PLUS (2D-SWE.PLUS) and viscosity PLUS (Vi.PLUS) measurements in a subject without thyroid pathology. Two colored maps are on display: the upper map corresponds to the 2D SWE evaluation, color-code blue corresponds to soft tissues, while red depicts increased stiffness. The viscosity map is displayed in the lower half of the image. Colors close to yellow-white describe high viscosity, while red illustrates low viscosity values. The quantitative 2D-SWE.PLUS results, expressed by a number (kPa) and of Vi.PLUS (Pa.S) are in this case displayed in the upper right corner of the image. The Q-Box measurements include the mean, median, minimum, maximum, and standard deviation (SD) for 2D-SWE PLUS, the mean, median and SD for ViPLUS, along with the diameter, depth of the QBox, and the Stability Index (SI) for the measurement.

The stability index, the diameter, and depth of the QBox are displayed on the screen for each measurement, together with the mean (Mean), median (Med) and standard deviation (SD) for ViPLUS and the Mean, Med, minimum (Min), Maximum (Max) and SD for 2D SWE PLUS (see Figure 1).

### 2.3. Statistical Analysis

For the statistical analysis, MedCalc V19.4 (MedCalc Software Ltd., Ostend, Belgium) was used. Descriptive statistics were applied for the clinical, demographic, anthropometric, and US findings. The numerical variables’ distribution was established using the Kolmogorov–Smirnov test. Numerical variables with normal distribution were presented by mean and standard deviation, whereas variables with non-normal distribution were presented as median and range interval. Qualitative variables were represented as percentages and figures. Pearson’s correlation coefficient was determined in order to assess correlations between normally distributed data and Spearman rank correlation (rho) in order to assess correlations for data with non-normal distribution. In univariate and multivariate statistical analyses, the predictors from the regression equations have been accepted in line with a repeated, stepwise backward algorithm, so as to obtain the most suitable prediction model. For each predictive test, 95% confidence intervals (CI) were calculated; a *p*-value below 0.05 was considered as statistically significant.

## 3. Results

### 3.1. Baseline Characteristics

A number of 121 consecutive adult subjects with no known thyroid pathology underwent multimodal US measurements (US, 2D-SWE PLUS and ViPLUS) and were included in the study. Of these, valid measurements were obtained in 115 cases (95.1%) and the other 6 cases (4.9%) were excluded from the final analysis. According to BMI distribution, most patients were in the normal range (18.5–24.9) and almost half of the subjects (41.7%) were in the age group 26–40 years. The baseline characteristics of the group are summarized in Table 1.

### 3.2. Feasibility and Reproducibility of ViPLUS

Using both 2D-SWE PLUS and ViPLUS, valid measurements were obtained in 115/121 cases (95.1%). Out of the six cases with invalid measurements, four were due to lack of filling of the color-coded map (too little signal) and two were due to a value of the SI < 90%. In terms of BMI, the values were significantly higher for the group with unreliable measurements when compared with the group that obtained reliable measurements (24.02 ± 4.35 kg/m^2^ vs. 27.6 ± 4.5.75 kg/m^2^, *p* = 0.049). No significant differences were found for age mean values (34 ± 12.7 years vs. 42.5 ±14.7 years, *p* = 0.119).

The feasibility of the method was analyzed. This parameter is defined as the likelihood of acquiring valid measurements. In our analysis, a very good feasibility (95%) was obtained for the concomitant evaluation of 2D-SWE PLUS and Vi PLUS. 

The intraobserver reliability was also tested for the ViPLUS evaluation. The intraclass correlation coefficient (ICC) had a value of 0.8634 (95% CI: 0.817 to 0.900) for the RTL and 0.8568 (95% CI: 0.808 to 0.895) for the LTL, showing good reliability for the viscosity assessment by SuperSonic ViPLUS, which is a reproducible technique.

### 3.3. Vi PLUS Values in the Healthy Thyroid Cohort and the Influence of Subjects’ Characteristics

The results for thyroid 2D-SWE and viscosity measurements are presented in Table 2.

**Figure 2:**
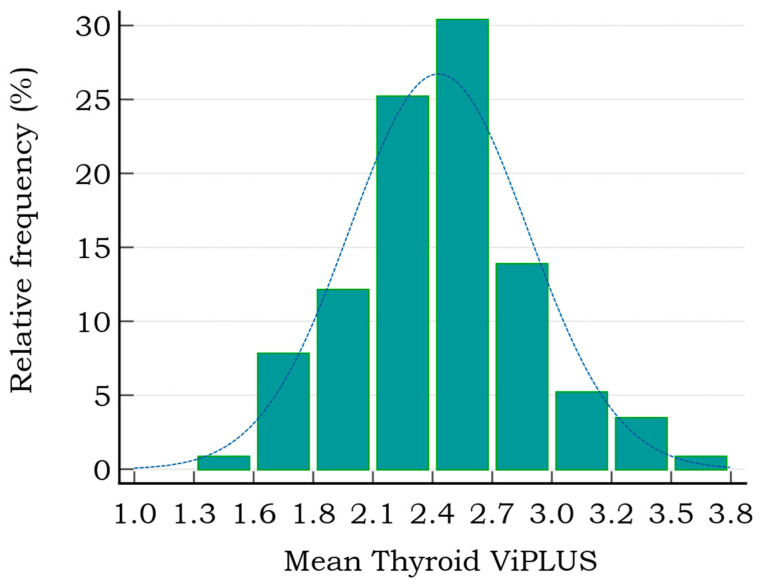
The distribution of thyroid mean Vi PLUS values in healthy subjects (range 1.3–3.8 Pa·s).

Mean values for normal thyroid viscosity ranged between 1.3 and 3.5 Pa·s, the distribution of the mean ViPLUS values is represented by a histogram in Figure 2. About 75% of Vi PLUS measurements were in the range (1.7–2.7).

**Figure 3:**
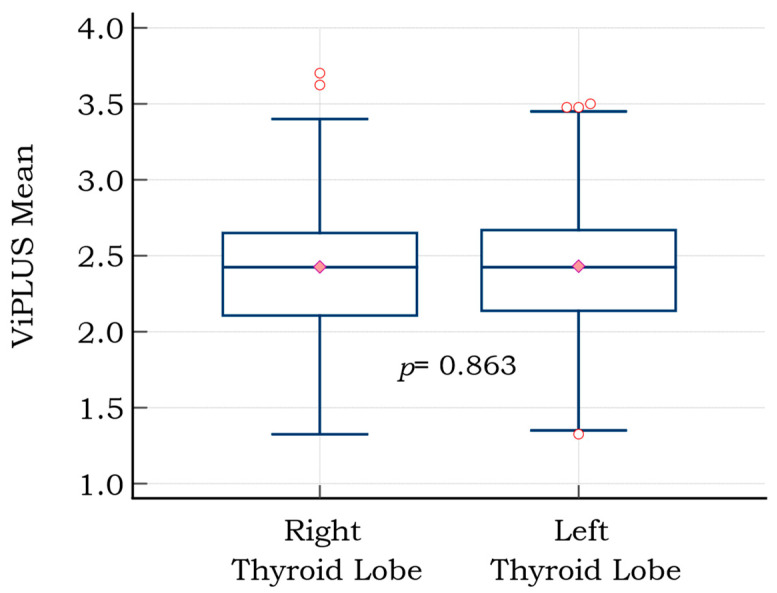
Graph comparison (box and whiskers) of mean viscosity plane-wave ultrasound (ViPLUS) values for the right and left thyroid lobes; the red circles are the graphical representation of outliers (extremely high, respectively extremely low values); the pink diamond marks the median value.

The mean ViPLUS values for both thyroid lobes were compared for each subject (median of 5 values for each lobe). A mean value of 2.42 ± 0.44 Pa·s was determined for the left thyroid lobe (LTL) measurements and a mean of 2.43 ± 0.44 Pa·s for the right. No significant differences were found between the left (LTL) and right thyroid lobe (RTL) (Figure 3). 

The means between the right and left lobe values for both 2D-SWE PLUS and ViPLUS for each patient were then included in the final analysis. According to age subgroups, the lowest ViPLUS values were obtained for patients over 40 years and the highest values were obtained for adults between 25–40 years, although differences between age groups were not statistically significant. For SWE, the relations were inverse, lowest values were observed for the group between 26–40, but age-group differences did not reveal statistical significance for this parameter either. The US-based measurements for elasticity and viscosity according to age subgroups are presented in Table 3.

Most of the patients in our group (71.3%) were female. Still, we did observe some differences in viscosity values between female and male gender: 2.38 ± 0.39 vs. 2.54 ± 0.46 (*p* = 0.06). 

In terms of BMI, no significant differences were found neither between the mean values for ViPLUS in overweight patients or patients with normal weight: 2.54 ± 0.37 Pa·s vs. 2.49 ± 0.43 Pa·s, (*p* = 0.663) nor for SWE: 12.71 ± 3.27 kPa vs.13.88 ± 3.25 kPa (*p* = 0.199).

**Figure 4:**
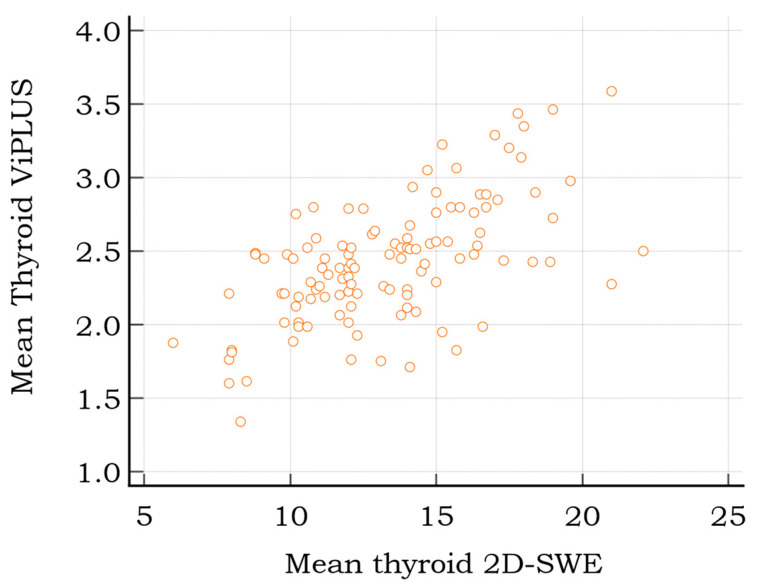
Scatter diagram displaying the relationship between 2D SWE and Vi PLUS thyroid measurements for healthy subjects.

A strong, positive correlation was found between 2D-SWE PLUS values and thyroid ViPLUS (r = 0.608, *p* < 0.0001); the data are represented as a scatter diagram in Figure 4**,** with the SWE measurements on the *x* axis and the ViPLUS data on the *y* axis.

We verified possible correlations between 2D SWE, the respective Vi PLUS measurements and age, gender, BMI and depth of the QBox, but no statistical significances were found (see Table 4).

In univariate regression analysis, thyroid stiffness values by 2D-SWE PLUS (*p* < 0.001) were associated with the ViPLUS values, but no association was obtained for age (*p* = 0.5127), BMI (*p* = 0.8563), depth of the measurements (*p* = 0.3088), nor for gender (*p* = 0.0566) and ViPLUS. Further, multivariate regression was employed to analyze the independent parameters associated with the ViPLUS values. A regression model was built according to a forward stepwise method. The best model was appreciated by using the Akaike Information criteria (AIC). The final model that included only thyroid stiffness values provided by 2D-SWE PLUS (*p* < 0.0001) was associated with Vi PLUS values.

## 4. Discussion

Elastography has gained increasing popularity as a medical imaging technology since the 1980s. The underlying premise of the various elastography techniques is that soft tissues are more easily deformed than stiff structures are, and that these differences are measurable [12,25]. This traditional viewpoint is changing, though, as more attention has recently been paid to the complex structures that soft tissues display, emphasizing the significance of strongly nonlinear hyperelastic, poroelastic, and viscoelastic behavior in addition to only elastic properties [21]. 

There is evidence linking changes in tissue viscosity to the degree of inflammation [22]. The dispersion effect is not taken into account by the majority of current ultrasonic SWE techniques. The potential of this important novel biomarker has led to the release of new imaging tools, initially created for liver assessment. These new imaging techniques, created up to the present by Supersonic Imagine and Canon, incorporate the algorithmic characteristics of tissue viscosity. Establishing the typical soft tissue viscosity value is the first step in verifying this novel approach. 

Our results show that in normal subjects, thyroid viscosity PLUS values range between 1.33 and 3.58 Pa·s, with a mean value of 2.42 ± 0.41 Pa·s. As a result, Vi PLUS values of around 2.4 Pa·s can be interpreted as being representative of normal thyroid, free of fibrosis or inflammation. Other studies have also investigated ViPLUS in superficial organs and a mean value of 2.13 ± 0.23 Pa·s was found for the parotid gland (PG) and of 2.44 ± 0.35 Pa·s for the submandibular glands (SMG) [26]. Interestingly, thyroid viscosity found by our study and submandibular gland viscosity values found by the above-mentioned study are highly similar. The echogenicity of the SMG and PG has previously been considered as reference for the echogenicity of normal thyroid tissue [27], but elasticity or viscosity values were not yet compared. 

Liver viscosity was firstly investigated as a mechanical parameter by Deffieux et al. (2015) as potential predictor of disease activity, steatosis or fibrosis [28]. Their results show that viscosity was only a weak predictor of disease activity and steatosis levels and that it had a poorer predictive value for fibrosis, compared to stiffness. Normal liver viscosity values on the same device, using the same technique, were found by our group to be around 1.59 Pa·s [29]. In contrast with our findings, liver values of ViPLUS did increase with age but the concept of “inflammaging” described for the liver—the increase in necroptosis associated with age—is not characteristic for thyroid tissue [30,31]. 

According to Sugimoto et al. (2018) [22], the degree of lobular inflammation was highly related to dispersion slope, and the stage of fibrosis was strongly correlated with shear-wave speed. Viscosity surpassed elasticity in predicting the degree of necroinflammation, whereas elasticity outperformed viscosity in estimating the stage of fibrosis [22]. This statement needs to also be verified for other parenchymatous organs, such as the thyroid gland, and could potentially be truly helpful in distinguishing thyroid aggression or inflammation, such as subacute thyroiditis from thyroid fibrosis. Higher Vi PLUS liver levels have been reported in patients with COVID-19 pulmonary injury compared to COVID-19 patients without pulmonary injury, according to a recently released study [32]. In another study [33], the viscosity parameter was measured for focal liver lesions (FLL) using the Canon Aplio device that uses the shear wave dispersion slope, which is directly correlated to viscosity. Significantly higher mean values of the viscosity parameter were detected for malignant FLLs than for benign FLLs (14.79 3.15 versus 13.36 2.76 m/s/KHz). 

The same technique for measuring tissue viscosity was used in a recent study that evaluated the US-based methods in the assessment of renal allograft. A positive correlation was established between eGFR and ViPLUS values, but also between 2D SWE PLUS and ViPLUS [34]. In our study, thyroid ViPLUS values also revealed a moderate positive correlation with the 2D-SWE measurements.

The applications of viscosity measurements are limitless, although the US-based techniques used in this study have not yet been used in other organs’ characterization. According to McFarlin et al. [35], cervical ultrasound attenuation, which is connected to compressional viscosity but not shear viscosity, may help to identify women who are at risk of spontaneous preterm birth. For prostate, one of the few methods employed is shear wave dispersion ultrasound vibrometry (SDUV), but the results are also encouraging [36]. 

While it is unlikely that non-invasive US-based techniques such as ViPLUS and 2D-SWE PLUS will ever be able to completely replace the gold standard for diagnosis, such as fine-needle aspiration (FNA) as in the case of thyroid nodules and FNA or antibody measurements for autoimmune thyroid disease, they are easier, more rapid and accessible methods for a quick lead of diagnosis in unclear cases and are also useful in assessing the evolution of the disease over time. Another advantage is that thyroid ViPLUS measurements have strong intraoperator agreement, according to our study. The viscosity values were stable throughout the cohort; they were not influenced by BMI, age, gender or depth of the ROI. 

One important limitation of our study was that the curvilinear transducer was employed, since the ViPLUS module could only be used with this kind of transducer at the time of study inclusion. However, the images achieved an SI of over 90%, which confirmed the validity of measurements. The implementation of the ViPLUS software on the linear probe is expected for a more exact characterization of superficial structures.

## 5. Conclusions

ViPLUS is a cutting-edge ultrasound-based method that is reproducible and feasible for the evaluation of thyroid parenchyma. Our results are encouraging for the use of ViPLUS, along with 2D-SWE, for thyroid evaluation in clinical practice; they also open the way for further studies evaluating the usefulness of this ultrasound tool in detecting thyroid pathology. 

## Figures and Tables

**Table 1:** Descriptive data of the healthy subjects with ViPLUS reliable measurements.

Parameter		Value
Total number		115
Gender	Male	33 (28.7%)
	Female	82 (71.3%)
Mean BMI		24.02 ± 4.35 kg/m^2^
Mean age		34 ± 12.7 years
Age groups (number of subjects)	18–25	39
	26–40	48
	>40	28

Data are presented as numbers and percentages or mean ± standard deviation. BMI = body mass index, 2D-SWE PLUS = two-dimensional shear-wave elastography (SuperSonic Imagine).

**Table 2:** US-based two-dimensional SWE and viscosity measurements of thyroid tissue in healthy subjects.

US-Based Parameter		Value
Mean 2D-SWE PLUS (kPa)	Mean ± SD	13.34 ± 3.2
	Min	6.0
	Max	22.1
ViPLUS (Pa·s)	Mean ± SD	2.42 ± 0.41
	Min	1.33
	Max	3.58
Depth		1.6 ± 0.26 cm

2D-SWE PLUS = two-dimensional shear-wave elastography; ViPLUS = ShearWave Viscosity Plane-wave UltraSound (SuperSonic Imagine); SD = standard deviation; Min = minimum value; Max = maximum value.

**Table 3:** Normal thyroid tissue values for 2D SWE and ViPLUS according to age subgroups.

Age Subgroup	Mean 2D-SWE PLUS (kPa)		Mean ViPLUS (Pa·s)	
18–25	13.6 ± 3.2	*p* = 0.500	2.38 ± 0.38	*p* = 0.391
26–40	12.9 ± 2.9	*p* = 0.310	2.49 ± 0.43	*p* = 0.127
>40	13.57 ± 3.64	*p* = 0.661	2.36 ± 0.43	*p* = 0.376

2D-SWE PLUS = two-dimensional shear-wave elastography; ViPLUS = ShearWave Viscosity Plane-wave UltraSound (SuperSonic Imagine).

**Table 4:** Correlations between 2D SWE, the respective Vi PLUS measurements and patient or measurement characteristics.

		BMI	Depth	Age	Gender	Mean SWE
Mean SWE	Correlation coefficient	−0.195	0.018	0.008	0.008	-
	Significance Level p	0.1498	0.8466	0.9283	0.9243	
	n	115	115	115	115	
Mean ViPLUS	Correlation coefficient	0.025	0.096	−0.101	0.178	0.608
	Significance Level p	0.8563	0.3088	0.2842	0.0566	<0.0001
	n	115	115	115	115	115
